# Periodic-peristole agitation for process enhancement of butanol fermentation

**DOI:** 10.1186/s13068-015-0409-6

**Published:** 2015-12-23

**Authors:** Meng-lei Xia, Lan Wang, Zhi-xia Yang, Hong-zhang Chen

**Affiliations:** State Key Laboratory of Biochemical Engineering, Institute of Process Engineering, Chinese Academy of Sciences, No. 1 Beiertiao, Zhongguancun, Haidian District, Beijing, 100190 China; University of Chinese Academy of Sciences, No. 80 Zhongguancun East Road, Haidian District, Beijing, 100039 China; College of Mathematics and System Science, Xinjiang University, No. 14 Shengli Road, Urumchi, 830046 China

**Keywords:** Periodic-peristoleagitation, Butanol, Hydrodynamic analysis, Metabolic flux analysis, Enhancement mechanism of agitation, Rational enhancement strategy

## Abstract

**Background:**

Mass transfer plays an important role in determining the efficiency of the biofuel conversion. However, adverse effect of shear stress from traditional agitation inhibits the cell growth and production of biofuels. How to enhance the mass transfer with less adverse effect is considered as one of the important bioengineering issues.

**Results:**

In this study, a novel agitation type, named periodic-peristole was applied to butanol fermentation with *Clostridium acetobutylicum* ATCC 824. Meanwhile, the enhancement mechanism was studied. Initially, the fermentation performance of periodic-peristole agitation was compared with the traditional *Rushton* impeller and stationary cultivation. Result showed that the biomass, butanol and total solvent in periodic-peristole group (PPG) was enhanced to 1.92-, 2.06-, and 2.4-fold of those in the traditional *Rushton* impeller group (TIG), as well as 1.64-, 1.19- and 1.41-fold of those in the stationary group (SG). Subsequently, to get in-depth insight into enhancement mechanism, hydromechanics analysis and metabolic flux analysis (MFA) were carried out. The periodic-peristole agitation exhibits significant difference on velocity distribution, shear force, and mixing efficiency from the traditional *Rushton* impeller agitation. And the shear force in PPG is only 74 % of that in TIG. According to MFA result, fructose 6-phosphate, pyruvate, acetyl-CoA, oxaloacetate and α-ketoglutarate were determined the key nodes of cells in response to hydrodynamic mechanical stress. Based on such key information, rational enhancement strategies were proposed and butanol production was further improved.

**Conclusion:**

The agitation associated with three issues which resulted in significant changes in cell metabolic behaviors: first, a rebalanced redox status; second, the energy (ATP) acquirement and consumption; third, the tolerance mechanism of the cell for survival of solvent. Periodic-peristole agitation provides an answer to address a long-standing problem of biofuel engineering. Key information derived from current study deepens the understanding of agitation, which can guide the designment of new bioreactors and development of enhancement strategies for biofuel refinery.

**Electronic supplementary material:**

The online version of this article (doi:10.1186/s13068-015-0409-6) contains supplementary material, which is available to authorized users.

## Background

Biobutanol is one of the bioalcohols that have gained considerable attention in recent years. However, regarding the industrial production of biobutanol, much effort is still needed to improve the production titer, especially from the view point of lowering the production cost [1]. The higher butanol titer inevitably requires suitable process conditions [1, 2], among which agitation plays a critical role mainly for maintaining the solid–liquid suspension homogeneous to ensure good mass transfer in (nutrients) and out (metabolites) of the microbial cell [3]. It directly affects the substrate consumption and yield of fermentation end products, thus affecting the overall process economics.

Over the past few years, how to enhance butanol fermentation by agitation has been the subject of several studies. Doremus et al. [4] found that the agitation rate plays an important role in controlling the metabolism of *C. acetobutylicum.* Agitation favors butyric acid productivity during the acid phase and hinders the butanol biosynthesis in the solvent phase. Lamed et al. [5] showed that agitation can dramatically affect the level of dissolved hydrogen gas and solvent ratio in fermentations by *Clostridium thermocellum*. In most of the cases, the total effect of agitation on butanol is inhibition rather than promotion due to the adverse effect of agitation [4, 6]. Thus, new agitation type with less shear force is urgently required for the biofuel engineering [7], which can be achieved only through a full understanding of the undergoing agitation-associated mechanism.

Nowadays advantages in fluid mechanics enable us to gain a more in-depth insight into the complex effects of agitation processes. Models have been successfully exploited in determining the correlationship between agitation and fermentation process, such as maximum stable (equilibrium) drop size in intermittent turbulence [8, 9], hydrodynamic stress [10], eddy length [11], and energy dissipation [12]. At the same time, developments on metabolic flux analysis (MFA) also allows for the systematical elucidation of cellular behaviors [13–16]. MFA is a powerful tool that allows addressing the study of production systems in a comprehensive manner, considering their specific metabolic capabilities, requirements and culture conditions [17]. Results obtained from MFA can help to interpret current results and guide future experiments leading to an enhanced yield of the targeted product. Therefore, the combination of fluid mechanics and MFA provides the possibility for understanding the association mechanism between cell metabolism and agitation at the extracellular and intracellular levels.

In this study, a novel agitation model named *periodicperistalsis* was developed for butanol fermentation. Firstly, the fermentation performances of *periodicperistalsis* were compared with existing methods. Then, the agitation effect and cellular physiology states were investigated using fluid mechanics analysis and MFA. Finally, the association mechanism between cell metabolism and agitation was discussed and the rational enhancement strategy was performed.

## Result and discussion

### Periodic-peristole agitation for enhancement of butanol fermentation

The periodic-peristole agitation came from the illumination of stomach and intestine. By expanding and contracting periodically, stomach and intestine can mix the food and digestive juices (mostly digestive enzymes) efficiently. This process is usually called “*periodic peristalsis*”. In this study, we applied this agitation type into the butanol fermentation process. At the same time, the traditional *Rushton* impeller agitation and stationary cultivation were set as references for comparison (The configuration comparison between the periodic peristalsis agitation and the traditional *Rushton* impeller agitation is given in Additional file 1: Figure S1). The fermentation profiles of the three groups are given in Fig. 2. Results showed that cell growth, substrate utilization and product biosynthesis exhibited significant differences among the three groups (the student’s *t* test result is given in Additional file 2: Table S1). Time course profiles of key fermentation parameters of H_2_ and CO_2_ from the periodic-peristole group (PPG), the traditional *Rushton* impeller group (TIG) and the stationary group (SG) are given in Additional file 3: Figure S2.

**Fig. 1:**
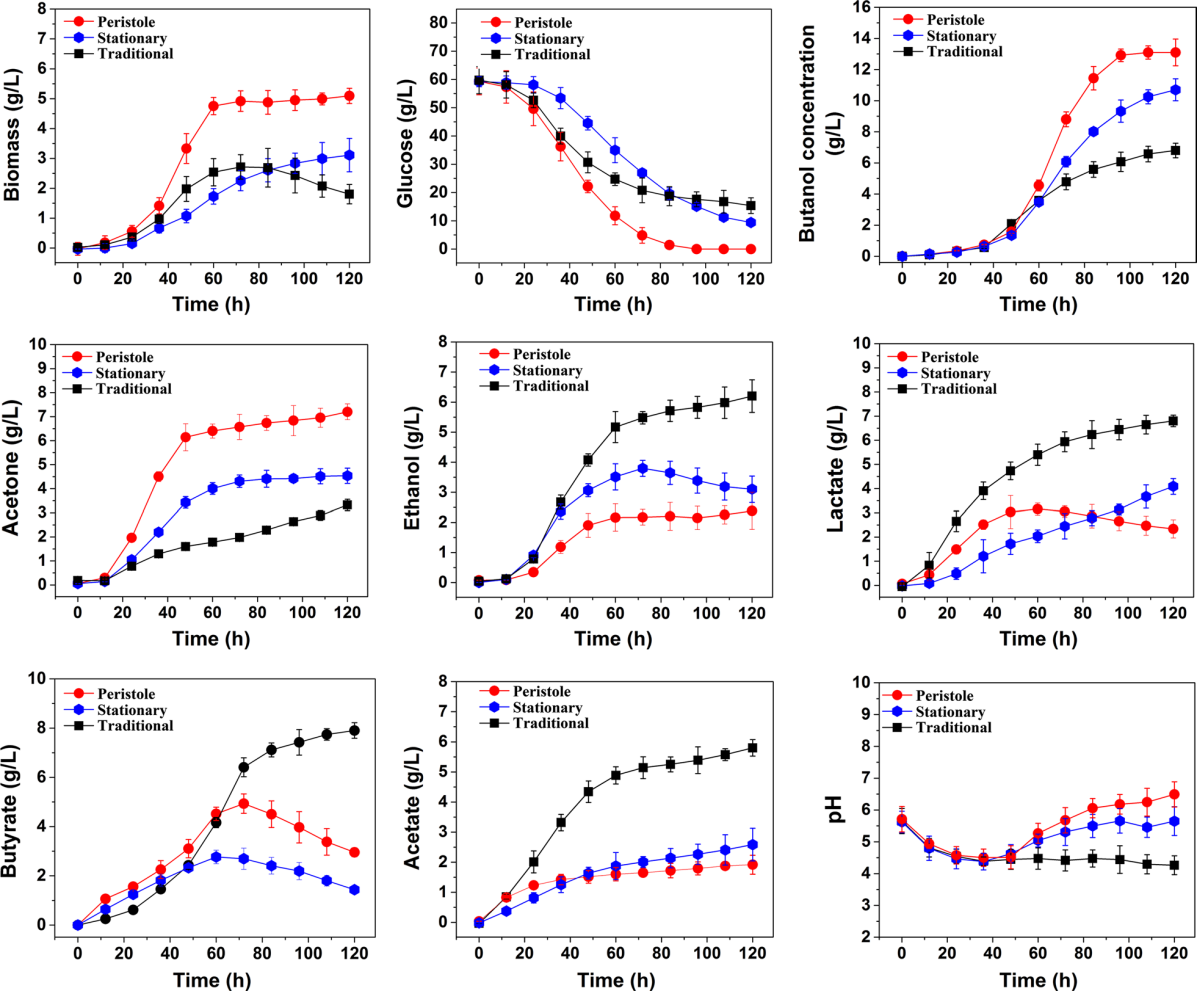
Time course profiles of key fermentation parameters (dry cell weight, glucose, butanol, acetone, ethanol, lactate, butyrate, acetate, and pH) from PPG, TIG and SG. PPG represents periodic-peristole group, TIG represents traditional *Rushton* impeller agitation group, and SG represents stationary culture group

The fermentation process of ABE can be divided into two phases: the acid-producing (or acidogenic) phase and the solvent-producing (or solventogenic) phase [15]: the acidogenic phase was observed during the first 60 h when cell biomass was rapidly produced. During this period, biomass in PPG and TIG was 2.75- and 1.47- fold of that in SG, suggesting agitation could promote cell growth in this phase. During 96–120 h (the solvent—producing phase), however, biomass in the TIG decreased sharply, and obvious cell autolysis was observed. It should be noted that butanol concentration in TIG was only 6.4 g/L (2.1 g/g biomass), which is much below the threshold (16.2 g/L) of cell tolerance [2]. Therefore, the cell autolysis in TIG was probably mainly the result of hydrodynamic mechanical stress. Strain in PPG consumed the most glucose in the media at 110 h. However, there were 9.4 g/L and 15.3 g/L glucose remained in TIG and SG until the end of the fermentation (120 h), indicating periodic peristalsis could promote glucose utilization. At the same time, production profiles under different agitation types were quite different: just as Fig. 1 shown, PPG produced more butanol and acetone while TIG had higher concentration of butanol, lactate, butyrate, and acetate.

### Hydrodynamic analysis of periodic-peristole agitation

**Fig. 2:**
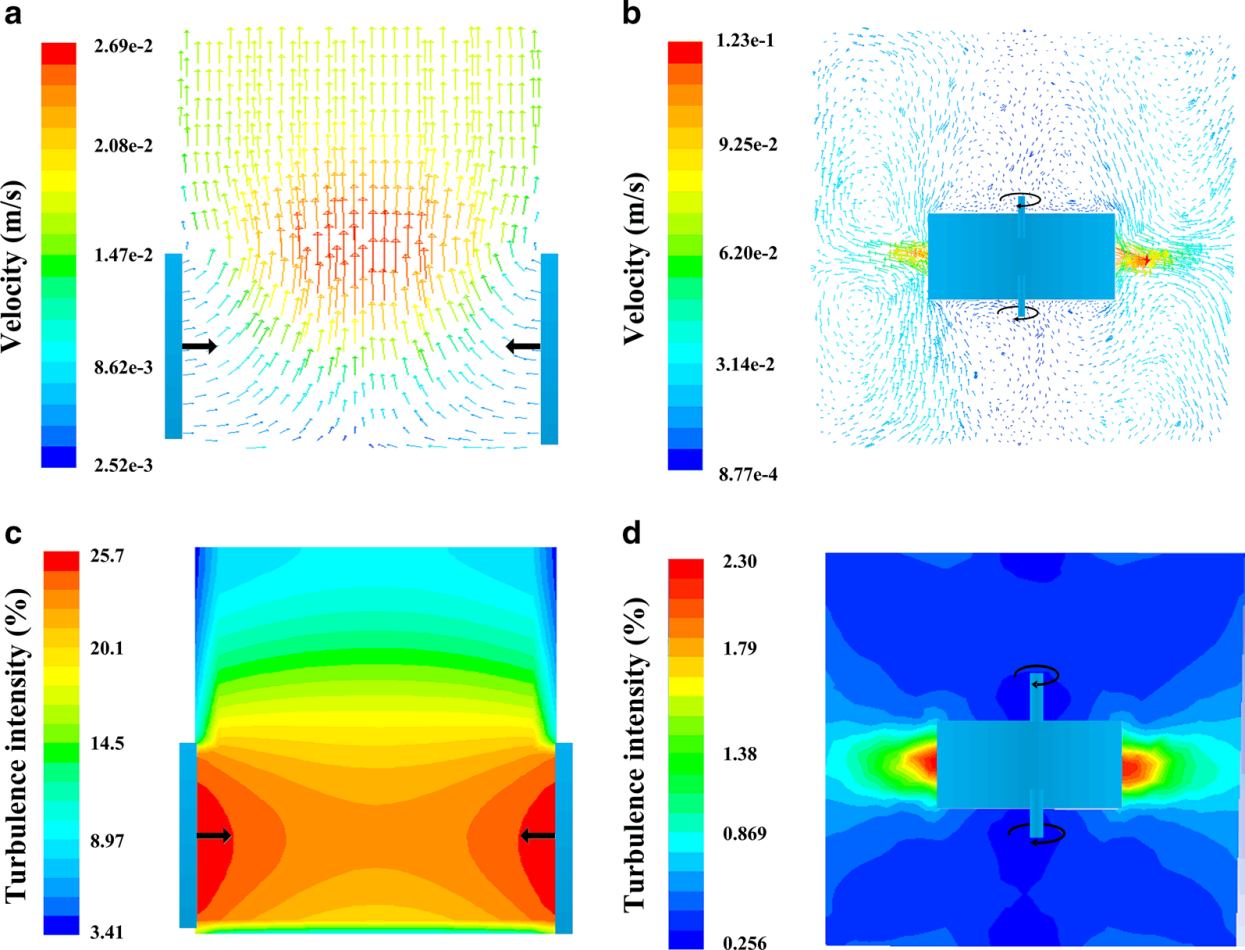
The comparison of agitation characteristics between PPG and TIG. **a** velocity distribution within PPG; **b** velocity distribution within TIG; **c** the turbulence intensity distribution within PPG; **d** the turbulence intensity distribution within TIG. PPG represents periodic-peristole group, TIG represents traditional *Rushton* impeller agitation group, and SG represents stationary culture group

Hydrodynamic characteristic of the fermentation system is one of the external factors responsible for the metabolism rate of cells [3, 4, 9]. It has been discussed for a long time to have large influence on biological process [18], cell viability, and product titers [19]. Therefore, hydrodynamic characteristic analysis was applied to PPG, with TIG as the reference for comparison. The results are given in Fig. 2 and Table 1.

**Table 1:** Comparison of the geometrical details of the rectors used and the turbulence parameters during the fermentation process

	Traditional *Rushton* impeller	Periodic- peristole reactor
Reactor volume	4 L	4 L
The tank diameter, D	0.14	0.14
The tank height (m), H	0.25	0.25
Mixed mode	*Rushton impeller*	Peristole
Main Flow type	Axial and radius direction	Axial direction
Impeller diameter (m), d	0.03	–
Number of blades	1	–
Power input (Pm)	23 W	18 W
Agitation rate (rpm)	55	–
Shrinking rate (m/s)	–	0.005
Eddy length (m)	9.54 × 10^−5^	1.28 × 10^−4^
Velocity range (m/s)	0–0.173	0–0.0269
The maximum turbulence intensity (%)	2.3	25.7
Mixing time (s)	120	95

#### Mixing characteristics

Figure 2 shows the computational fluid dynamics (CFD) simulation results of the two agitation types. Figure 2a indicates that the velocity distribution in PPG formed a large circulation in the bioreactor, which flowed from the bottom to the top (When the shrinking wall extended, the circulation ran reversely). The velocity values distributed uniformly both in axial and radius directions inside the whole bioreactor without showing a large difference, except the small ranges near the shrinking wall. The velocity distribution within TIG (shown in Fig. 2b) was quite differentiated: the velocity near the impeller was very high, which was almost 4.5-fold of the maxium velocity in PPG. This range is where cell suffered the most severe force damages [20]; The whole volume of the bioreactor was divided into different circulation fields, which decreases the mixing efficiency [21].

Figure 2c, d compare the turbulence intensity between the two biorectors. It can be found that most part in PPG had high turbulence intensity, which was almost tenfold of that in TIG. The turbulence intensity is a scale characterizing turbulence expressed as a percent. It has been reported that the turbulence intensity is related closely to the mixing effect. The larger the turbulence intensity is, the better the mixing effect is and the faster the mixing process is [22]. Mixing time experiment showed that the mixing of PPG was 95 s and that of TIG was 120 s, which proved that PPG was better in mixing efficiency than TIG.

#### Shear stress

**Fig. 3:**
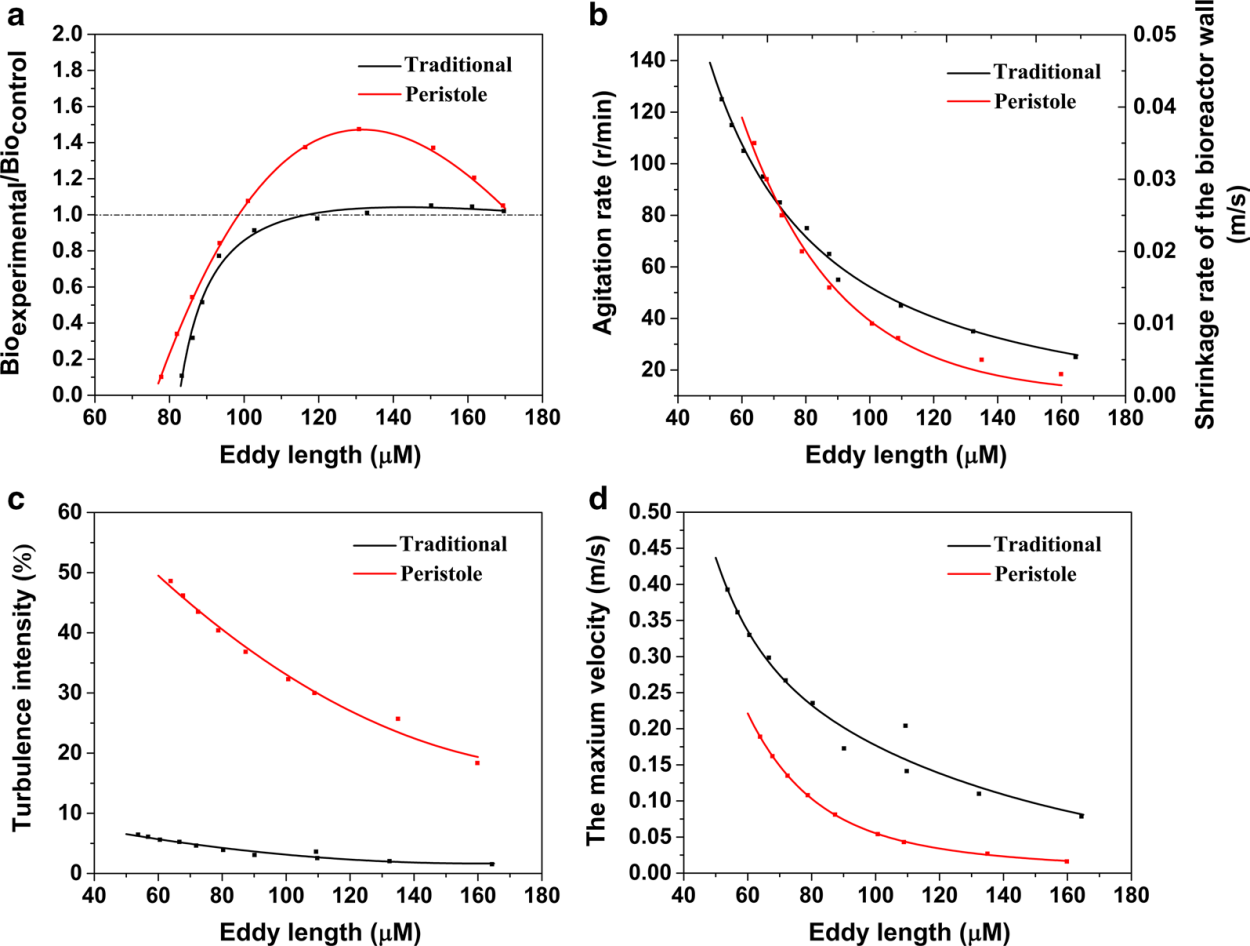
Hydrodynamics parameters in periodic-peristole agitation group and traditional *Rushton* impeller group. **a** shows the relationship between eddy length and cell growth enhancement rate. Cell growth enhancement rate was calculated by dividing biomass in the reference group (stationary culture group) by that in the experimental group at 120 h; **b** shows the relationship between eddy length and agitation velocity; **c** shows the relationship between eddy length and turbulence intensity; **d** shows the relationship between the eddy length and the maximum velocity

Eddy length is the most commonly used criterion to scale shear stress in bioreactor, which is based on the classical *Kolmogorov* model [11]. Figure 3a shows the relationship between eddy length and cell growth. As the Y axis of Fig. 3a, the term $\frac{{{\text{Bio}}_{\text{experimental}} }}{{{\text{Bio}}_{\text{control}} }}$ represents the ratio between biomass under different shear forces (experimental group) and without agitation (control group). It scales the promotion ($\frac{{{\text{Bio}}_{\text{experimental}} }}{{{\text{Bio}}_{\text{control}} }}$ > 1) or inhibition ($\frac{{{\text{Bio}}_{\text{experimental}} }}{{{\text{Bio}}_{\text{control}} }}$ < 1) effect of agitation on cell growth. Figure 3b shows the relationship between eddy length with agitation rate in TIG and peristole rate in PPG. It can be found that the inhibition effect of agitation on cell growth gets more severe with the decreasing eddy length. The critical point of the eddy length is 120 μm for *C. acetobutylicum* ATCC 824, below which the damage becomes visible. In our experiment, the eddy length in PPG is 128 μm and is 95.4 μm in TIG, indicating that the shear force in PPG is only 74 % of that in TIG.

Figure 3c, d show the relationship of eddy length with turbulence intensity and velocity. In the case of same eddy length, PPG always owns higher turbulence intensity (more than 20-fold of that in TIG in the whole range) and lower velocity (less than 60 % of that in TIG in the whole range). This characteristic makes periodic-peristole agitation different from the existing agitation methods. It enhances mass transfer by high turbulence intensity instead of high velocity, in other words, running under a mild condition. Therefore, this novel agitation causes less hydrodynamic damage to cells.

### Analysis of enhancement mechanism of periodic-peristole agitation using metabolic flux analysis

**Fig. 4:**
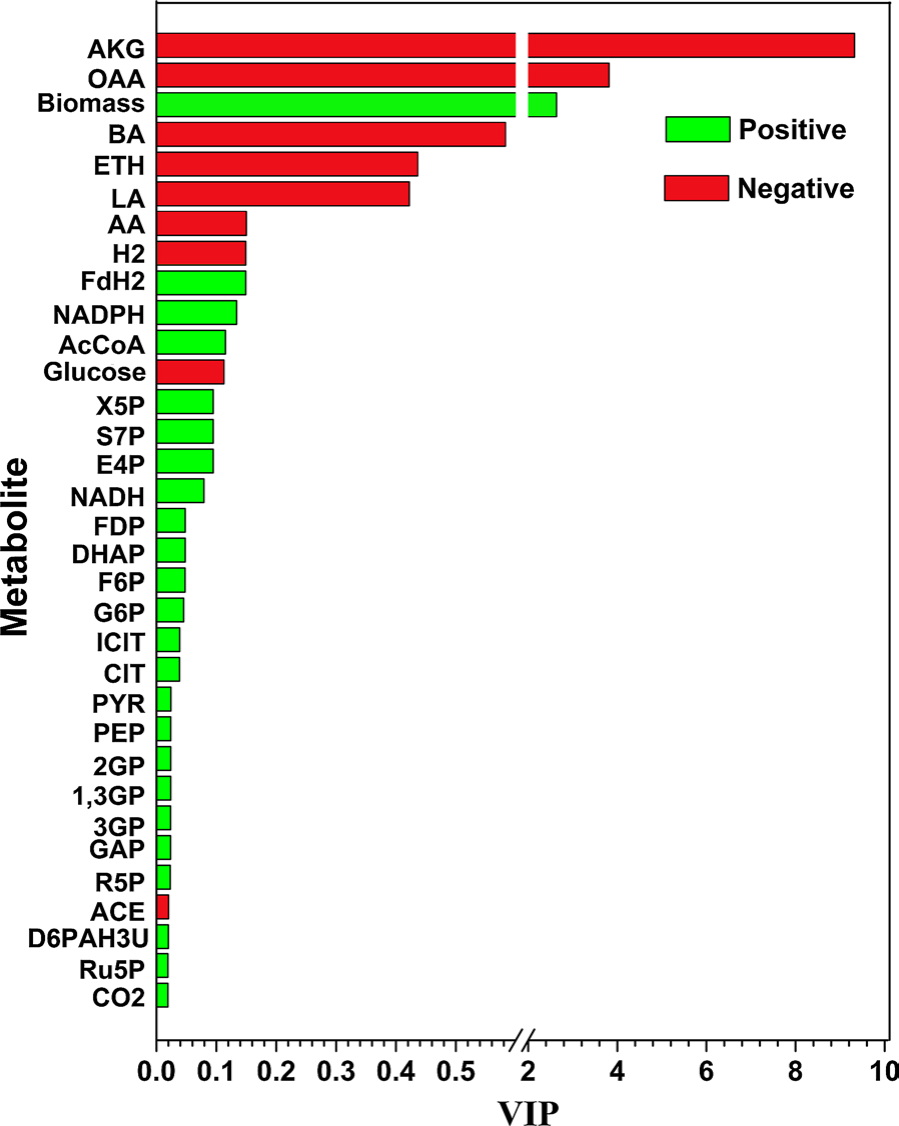
The VIP plots of the PLS model for butanol biosynthesis. *AKG* α-ketoglutarate, *OAA* oxaloacetate, *BA* butyrate, *Eth* ethanol, *LA* lactate, *AA* acetate, *AcCoA* acetyl-CoA, *X5P* xylulose-5-phosphate, *S7P* d-sedoheptulose-7-phosphate, *E4P* d-erythrose-4-phosphate, *FDP* d-fructose 1, 6-bisphosphate, *G6P* glucose 6-phosphate, *ICIT* isocitrate, *CIT* citrate, *PYR* pyruvate, *PEP* phosphoenolpyruvate, *2GP* 2-phospho-d-glycerate, *1,3GP* 3-phospho-d-glyceroyl phosphate, *3GP* 3-phospho-d-glycerate, *GAP* glyceraldehyde-3-phosphate, *R5P* d-ribose-5-phosphate, *ACE* acetone, *D6PAH3U* d-arabino-6-phospho-hex-3-ulose, *Ru5P* d-ribulose 5-phosphate

To understand how cells respond to agitation, MFA was performed on the metabolic data from PPG, TIG and SG, respectively. TIG and SG were set as references. The reactions and metabolites of the MFA model are provided in Additional files 4 and 5. To identify which metabolites were closely associated with butanol production, partial least squares discriminant analysis (PLS) was performed on the metabolic data from the different groups [23, 24] (VIP plot for biomass and total solvent are given in Additional file 6: Figure S3). The variable importance of the projection plot (VIP) score for each metabolite is shown in Fig. 4. A higher VIP score implies that the metabolite plays a more important role in butanol biosynthesis [23]. In the following part, combined with the PLS results, the flux distribution at the representative time points (48 and 96 h) are discussed in details. Representative time points were determined as cells showed the highest cell growth rate at 48 h and the highest solvent production rate at 96 h (Fig. 1).

#### Embden-Meyerhof-Parnas (EMP) pathway

**Fig. 5:**
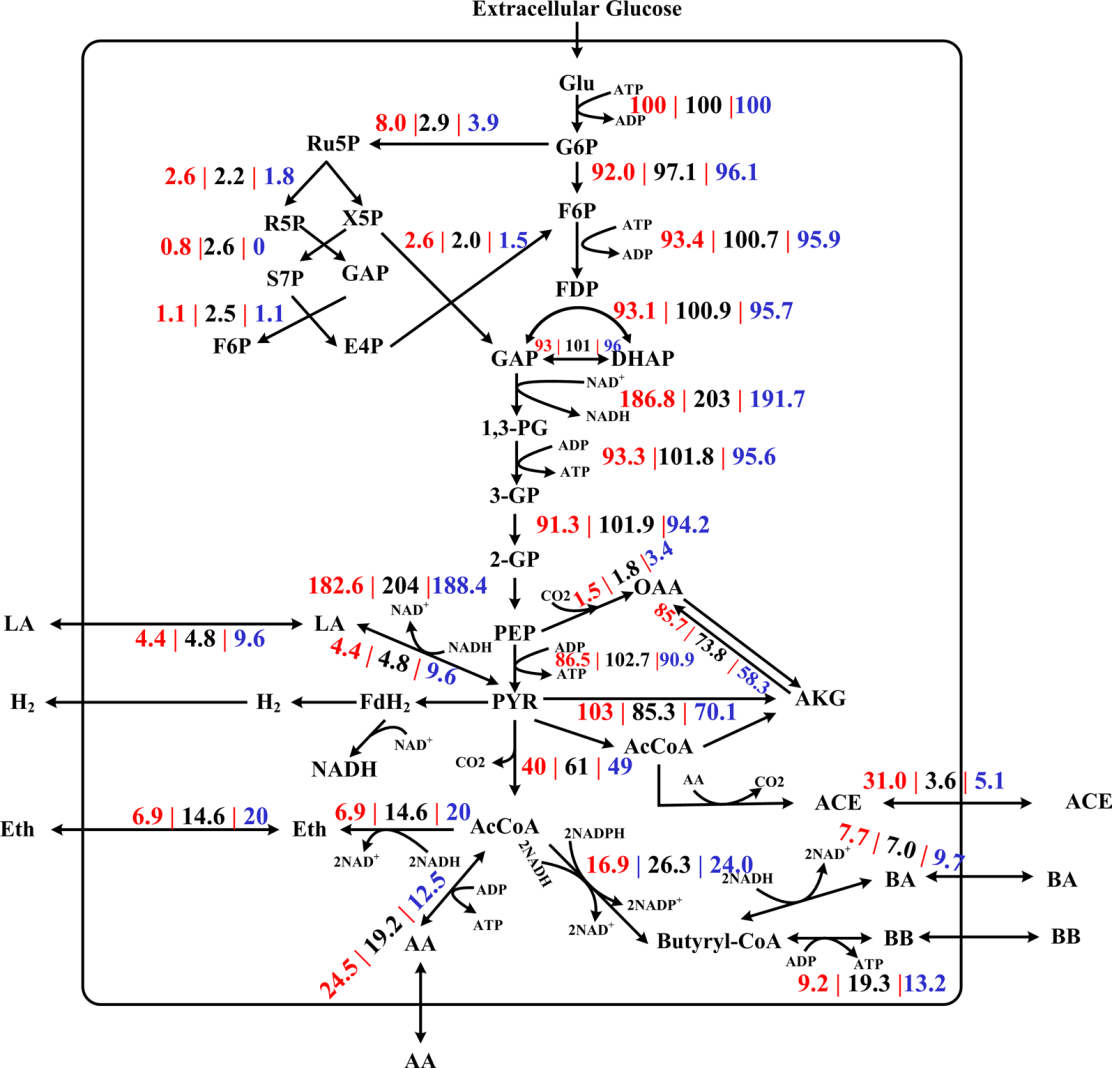
Flux distributions at flux distribution at 48 h from three cultivation models. The fluxes shown here have been normalized to make glucose uptake equal to 100 mM. The three numbers represent the carbon fluxes in PPG (in *red*), SG (in *black*) and TIG (in *blue*), respectively

**Fig. 6:**
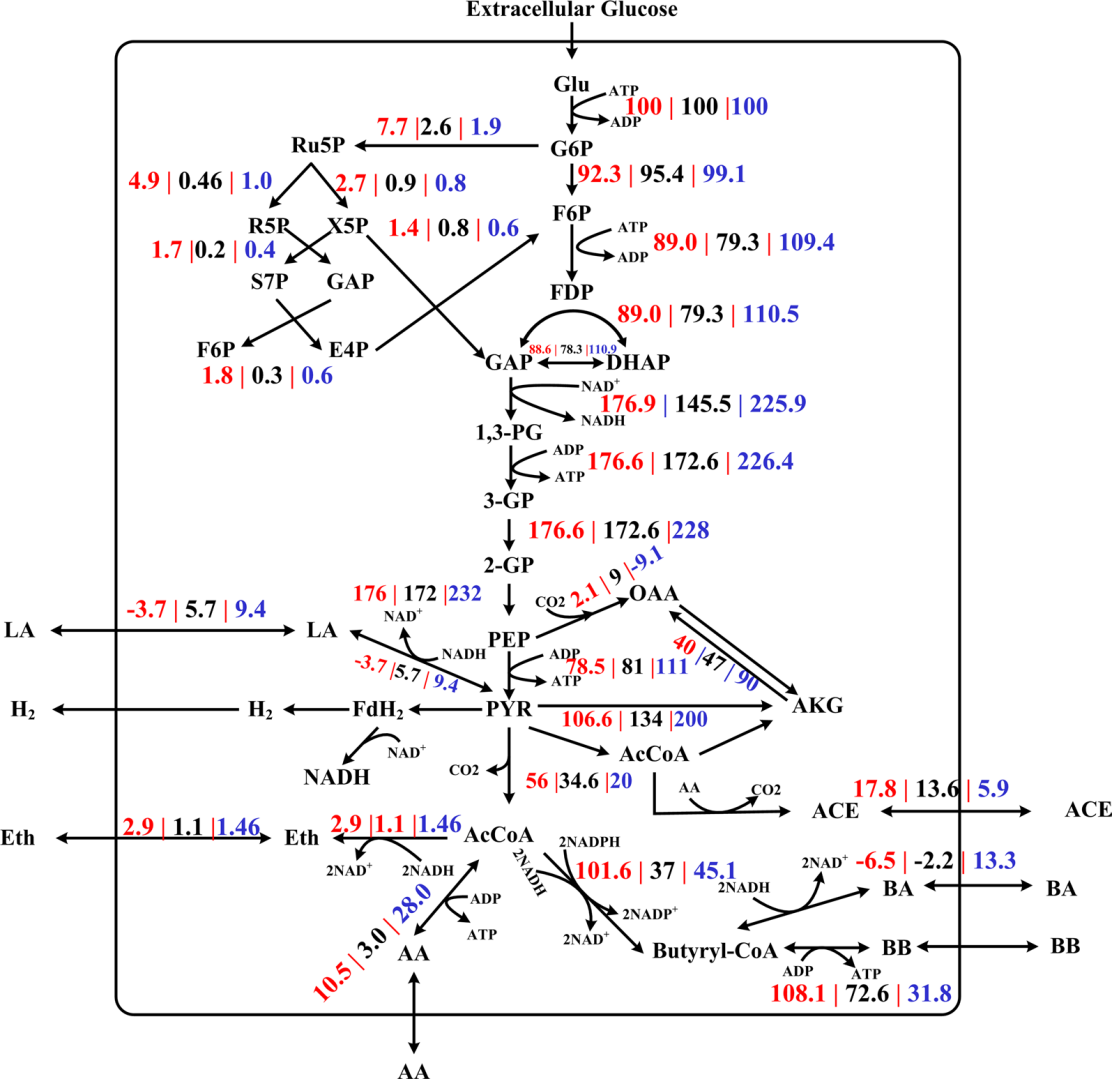
Flux distributions at flux distribution at 96 h from three cultivation models. The fluxes shown here have been normalized to make glucose uptake equal to 100 mM. The three numbers represent the carbon fluxes in PPG (in *red*), SG (in *black*) and TIG (in *blue*), respectively

Glucose was firstly converted into G6P and channeled into the EMP pathway and the PPP at this branch point. Then, 90 % of the glucose was further converted into pyruvate through the EMP pathway. At 48 h (Fig. 5), the EMP pathway showed a flux towards pyruvate in PPG and TIG to 84.2 and 88.5 %, respectively, compared with that in SG, suggesting that this flux contributed to increased cell growth through the PPP [25]; however, at 96 h the EMP pathway showed a flux towards pyruvate in PPG and TIG to 154.6 and 229.3 %, respectively, compared with that in SG, implying that the EMP pathway flux was significantly stimulated by agitation. The EMP pathway was the main source of ATP and NADH [25]. As shown in Fig. 6, the EMP pathway of cells in PPG and TIG generated 1.25-fold and 2.41-fold ATP, respectively, compared with that of SG at 96 h. Furthermore, the NADH generation rate from the EMP pathway of cells in PPG and TIG was 1.22-fold and 1.56-fold of, respectively, that of SG. These results indicated that cells produced more ATP and NADH through the EMP pathway following both types of agitation in the solvent—producing phase.

#### Pentose phosphate pathway (PPP)

PLS showed that metabolites in the PPP were important for butanol fermentation. At 48 h, the carbon flux of the PPP for cells in PPG and TIG was 2.75- and 1.34-fold of that in SG, respectively. This explains the high growth rate in PPG and TIG at 48 h, since cells in these groups produced more d-ribose-5-phosphate (R5P, the sugar backbone of nucleotides) to support rapid growth [25, 26] (The PLS analysis for cell growth is given in Additional file 6: Figure S3). At 96 h, the flux towards the PPP differed between the three groups: with PPG and TIG being 2.96- and 0.34-fold of, respectively, that of SG. The nonoxidative PPP flux generates the nucleotides required for DNA repair [27, 28], helping cells to tolerate butanol and solvent stress. Furthermore, on the metabolic network map (Figs. 5, 6), xylulose-5-phosphate (X5P), d-erythrose-4-phosphate (E4P), and d-sedoheptulose 7-phosphate (S7P) form a small loop that directs flux of the PPP flux back into the EMP pathway, potentially enhancing butanol synthesis by increasing the intercellular pyruvate pool.

#### Pyruvate metabolic pathway

After the EMP pathway, the flux enters the pyruvate metabolic pathway. Pyruvate is a key intermediate in cellular metabolic pathways. The glycolysis flux is converted to lactate or acetyl-CoA and CO_2_ through the pyruvate node. Acetyl-CoA is further converted to other end-products such as butyrate, butanol, and acetate. It is clear that phosphoenolpyruvate (PEP), pyruvate, and acetyl-CoA form three main key nodes in the flux distribution. PLS analysis showed that butyrate, lactate and acetate inhibited butanol production (Fig. 4).

The inhibition effect of butyrate might be due to the competition for the precursor, butyryl-CoA. As shown in Fig. 5, there are no significant differences (all P > 0.076) in the levels of butyrate among PPG, TIG and SG at 48 h. However, at 96 h, the flux towards butyrate biosynthesis (2 AcCoA + 2NADH → BA) in PPG and SG was −6.5 and −2.2 %, respectively, suggesting that butyrate was being absorbed for butanol synthesis [29]. By contrast, butyrate in TIG continued to be produced at a flux rate of 13.3 % (Fig. 6), competing for butyryl-CoA with butanol biosynthesis [30].

The difference in the flux distribution of acids (acetate, butyrate, and lactate) among the three groups may be strongly associated with the intercellular energy state. Acids producing pathways (acetate, butyrate, and lactate) and substrate-level phosphorylation form the main sources of ATP generation [15, 31, 32]. In the solvent—producing phase, the accumulation of butanol inhibits glucose uptake, thus inhibiting energy generation, which is compounded by an independent drop in intracellular ATP levels [2, 32]. To compensate for the ATP shortage, cells usually increase the flux towards acids synthesis.

#### TCA metabolism

In our MFA model, Oxaloacetate (OAA) and α-ketoglutarate (AKG) are the key metabolites that contribute to biomass production [13, 25]. Strikingly, they are listed as the most unfavorable metabolites for butanol synthesis (VIP of AKG and OAA were −9.3 and −3.8, respectively). In the solvent—producing phase, the flux towards AKG in PPG and TIG was 0.85- and 1.62-fold, respectively, that of SG at 96 h. The inhibition effect of these two metabolites is likely probably due to the consumption of acetyl-CoA competing with butanol biosynthesis. Based on previous studies [29], cells should cease to grow in the solvent—producing phase and distribute the flux towards butanol synthesis, just as seen with SG. Therefore, understanding this abnormality required further investigation into the metabolic network. It is known that the TCA cycle can provide the low redox potential of the internal anaerobic environment of *C. acetobutylicum*, as well as generating ATP [33]. Lee et al. [30] found that *C. acetobutylicum* M5 facilitates the biosynthesis of amino acids by altering the flux in the TCA cycle. Hence, the increased TCA flux in cells likely compensated the energy and amino acid pool.

#### Amino acid metabolism

**Fig. 7:**
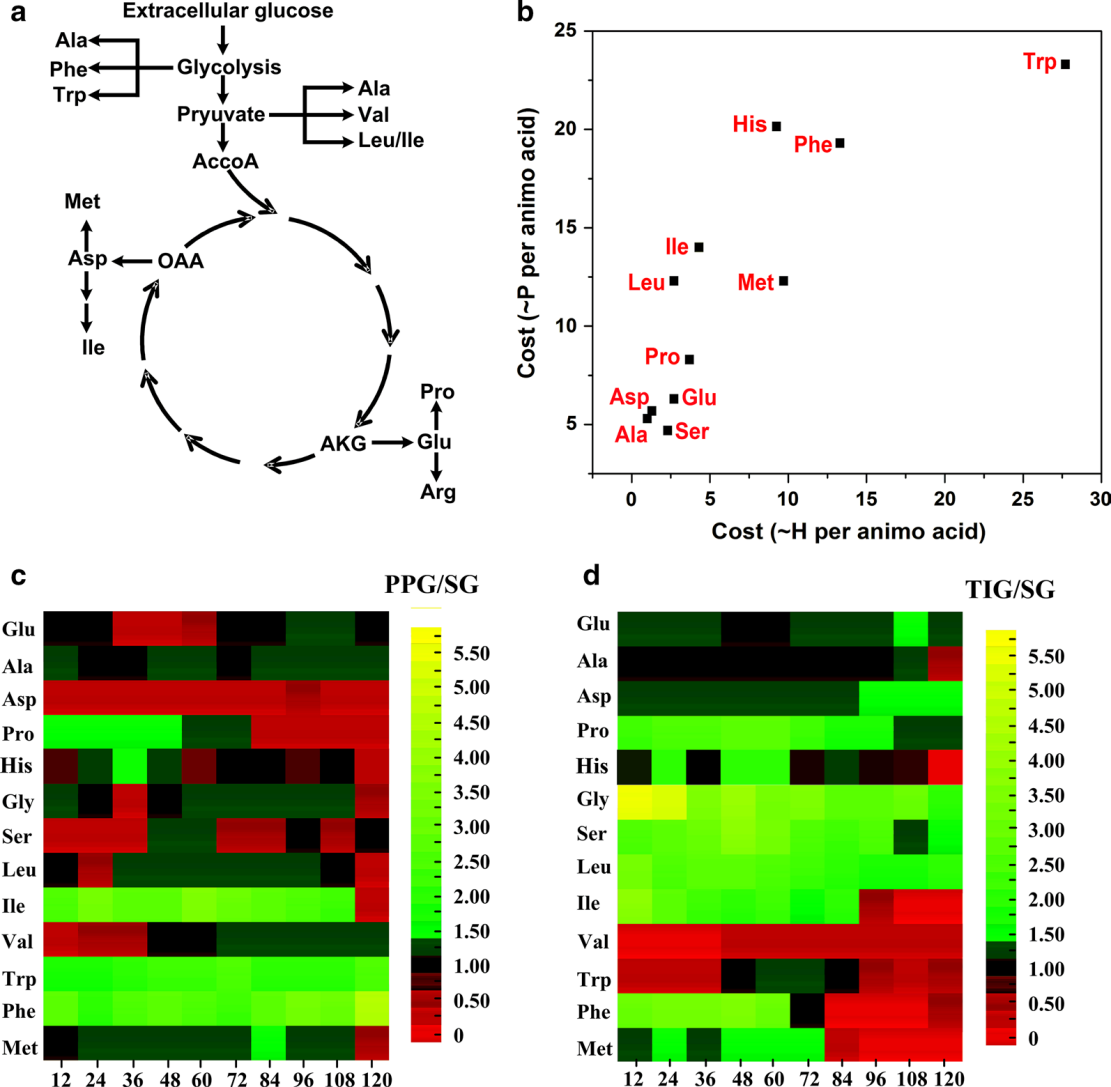
The comparison of the intracellular amino acids in periodic-peristole agitation group and traditional *Rushton* impeller group. **a** the synthesis pathways of the amino acids; **b** the energetic costs for amino acid biosynthesis; **c**, **d** the heat map visualizing the intracellular amino acids contents during fermentation from normal—and traditional groups. The *color code* indicates an increased (*green*) or a decreased (*red*) availability under the two conditions as compared to the reference process as indicated by the *color legend* as aside the *graph*. The full amino acid data set is given in Additional file 7. Availability for each amino acid was calculated as ratio of the concentration to that at reference group. *Glu* glutamate, *Ala* alanine, *Asp* aspartate, *Pro* proline, *His* histidine, *Gly* glycine, *Ser* serine, *Leu* leucine, *Ile* isoleucine, *Val* valine, *Trp* tryptophan, *Phe* phenylalanine, *Met*, methionine

Amino acids are key metabolites that reflect the intercellular energy status [23, 34, 35]. Studying on amino acids may aid our understanding of cell behavior. Therefore, intracellular amino acids from the three agitation modes were dynamically detected, as shown in Fig. 7.

Figure 7a shows the synthetic pathway for amino acids. From Fig. 7c, d, it was evident that in TIG the availability of some amino acids was exhausted during the solvent-producing phase, including isoleucine, tryptophan, and histidine. Given the energetic costs for amino acid biosynthesis (shown in Fig. 7b), these amino acids are the most energetically expensive ones. This further indicates that a reduced availability of metabolic energy might be involved in the reduced supply of the ‘expensive’ amino acids [35]. To validate our hypothesis, intracellular NADPH/NADP^+^, NADH/NAD^+^, and ATP were detected (as shown in Table 2). The intracellular concentrations of NADPH/NADP^+^, NADH/NAD^+^, and ATP at 96 h in TIG were 48.4, 19.5, and 37.6 %, respectively, of those in TIG at 48 h, and were 48.3, 40.0, and 57.6 %, respectively, of those in PPG at 96 h. These findings confirmed the ‘energy starvation’ status [23, 35] of cells in TIG during the solvent-producing phase.

**Table 2:** The dynamic changes of the important metabolites

Time (h)	36 h	48 h	96 h	108 h
NADH/NAD^+^	0.16 ± 0.056^a^	0.24 ± 0.079^a^	0.1 ± 0.026^a^	0.07 ± 0.012^a^
	0.12 ± 0.017^b^	0.17 ± 0.069^b^	0.06 ± 0.01^b^	0.05 ± 0.020^b^
	0.15 ± 0.037^c^	0.21 ± 0.092^c^	0.04 ± 0.02^c^	0.02 ± 0.011^c^
NADPH/NADP^+^	4.2 + 1.5^a^	10.32 + 1.8^a^	5.67 + 1.6^a^	2.21 + 1.83^a^
	3.45 + 0.98^b^	7.64 + 2.91^b^	3.46 + 1.79^b^	1.14 + 0.31^b^
	3.98 + 1.7^c^	5.65 + 1.79^c^	2.74 + 1.49^c^	0.67 + 0.38^c^
AcCoA^**^	1.37 ± 0.68^a^	2.10 ± 0.14^a^	1.90 ± 0.26^a^	0.07 ± 0.02^a^
	1.06 ± 0.42^b^	1.65 ± 0.11^b^	2.33 ± 0.17^b^	0.11 ± 0.03^b^
	1.34 ± 0.69^c^	2.17 ± 0.19^c^	1.84 ± 0.43^c^	0.03 ± 0.02^c^
ATP^**^	3.54 ± 0.82^a^	3.86 ± 0.23^a^	2.34 ± 0.14^a^	1.45 ± 0.15^a^
	4.27 ± 0.17^b^	2.88 ± 0.47^b^	1.93 ± 0.13^b^	1.11 ± 0.10^b^
	3.31 ± 0.21^c^	3.59 ± 0.56^c^	1.35 ± 0.09^c^	0.863 ± 0.16^c^
	27.2 ± 1.9^a^	28.5 ± 2^a^	39.8 ± 2.8^a^	25.3 ± 1.3^a^
Oleic acid**	25.7 ± 1.8^b^	25.7 ± 0.8^b^	32.4 ± 2.6^b^	27.2 ± 2.1^b^
	34 ± 2.4^c^	35.2 ± 1.4^c^	49.9 ± 2.5^c^	46.2 ± 3.6^c^
	23.6 ± 0.9^a^	35.1 ± 2^a^	31.8 ± 2.2^a^	28.3 ± 1.4^a^
Stearic acid**	25.5 ± 1.3^b^	36.7 ± 1.9^b^	29.4 ± 1.2^b^	29.9 ± 2.1^b^
	26.1 ± 0.8^c^	37.2 ± 1.6^c^	21.2 ± 1.9^c^	11.7 ± 1.6^c^
Linoleic acid**	35.9 ± 0.6^a^	15.7 ± 0.9^a^	18.3 ± 1.1^a^	19.4 ± 1.4^a^
	32.9 ± 1.0^b^	13.4 ± 0.4^b^	15.5 ± 0.5^b^	15.9 ± 0.8^b^
	32.8 ± 0.9^c^	13.8 ± 1.1^c^	21.6 ± 1.5^c^	23.5 ± 1.2^c^
Arachidonic acid**	42.1 ± 1.5^a^	38.5 ± 1.5^a^	30.1 ± 1.2^a^	23.3 ± 1.3^a^
	34.8 ± 2.1^b^	41.4 ± 2.1^b^	35.6 ± 1.3^b^	33.3 ± 1.8^b^
	45.4 ± 3.6^c^	46.2 ± 2.8^c^	43.1 ± 4.4^c^	41.5 ± 4.5^c^

** Stands for the special concentration at the corresponding time point (μmol/g); which is calculated by the concentration/biomass

^a^Stands for the periodic-peristole agitation group

^b^Stands for the Stationary culture group

^c^Stands for the traditional *Rushton* impeller

The ‘energy starvation’ status may strongly correlate with the solvent-resistance mechanism and agitation. In the presence of solvents, *Clostridia* increase branched chain amino acids and branched chain fatty acids to improve membrane structure stability, a process known as ‘*homeoviscous adaptation*’ [31, 36, 37]. Simultaneously, the hydrodynamic damage of the cell membrane caused by agitation also triggers the synthesis of tolerance protein [7, 38]. This process requires high quantities of ATP because amino acid synthesis is energetically expensive. [35]. To maintain a high-energy status, which is also one of the central requirements for cellular metabolism [39], cells have to rearrange their metabolism (acid biosynthesis in our study) towards enhanced ATP synthesis [23, 35, 40].

#### Fatty acid metabolism

Fatty acids have long been recognized as signaling molecules that have the capacity to trigger profound physiological responses [23, 38, 41]. Table 2 shows the dynamic profiling of fatty acids during the fermentation process. TIG possessed a high level of total unsaturated fatty acids, 0.15-, 0.3-, and 0.6-fold higher than that of PPG at 48, 96, and 108 h, respectively. However, the total amount of saturated fatty acids in TIG was lower than that of the other groups, being 106.3, 66.1, and 41.3 % of that in the PPG and 101, 72.1, and 39.1 % of that in the SG at 48, 96, and 108 h, respectively.

The cell membrane is a flexible structure composed of a lipid bilayer and proteins, and its fluidity is determined by the fatty acid composition. When under agitation, cells adjust their fatty acid metabolism to resist the adverse effects of shear force by improving cell membrane fluidity. Bhagyalakshmi et al. found that endothelial cells activate phospholipid turnover and enhance the biosynthesis of arachidonate under fluid shear stress [42]. Han, Yuan [38] demonstrated that *Axus Cuspidata* cells increase phosphatidic acid and phospholipase C to limit shear force damage. In our study, the high levels of unsaturated fatty acid in TIG may arise for a similar reason: during the acid—producing phase, shear forces may upregulate the synthesis of unsaturated fatty acids to increase cell membrane fluidity. Because suffering more serious shear force than the other two groups, cells in TIG own the highest content of unsaturated fatty acids. However, in the solvent—producing phase, the accumulation of solvents, especially n-butanol, began to disrupt the phospholipid bilayer of the cell [43–46], a phenomenon enhanced by shear stress. To exacerbate this, cells in TIG were unable to synthesize saturated fatty acids rapidly enough to inhibit the flow of organic solvent into the cell because of ‘energy starvation’, leading to autolysis. By contrast, in PPG, the hydrodynamic damage was not as pronounced as in TIG because the relatively higher energy status enabled cells to redistribute their flux and initiate the tolerance mechanism efficiently.

#### Association mechanism between agitation and cell metabolism

**Fig. 8:**
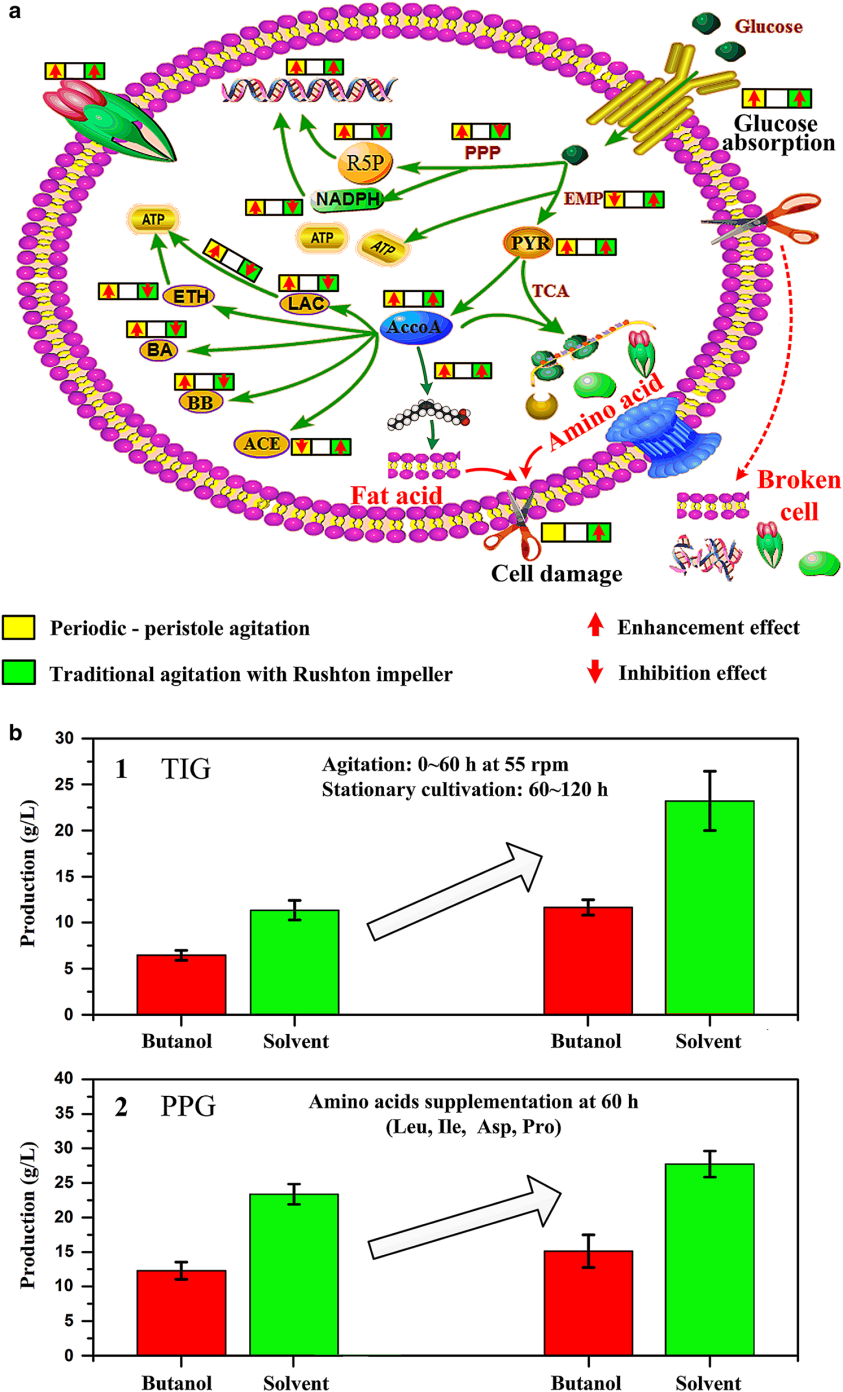
Effects of agitation on metabolic distribution of *C. acetobutylicum* and the corresponding enhancement strategy for butanol production. **a** summarizes the effects of agitation on cell metabolism. **b** shows the enhancement effects of rational strategies based on our supposed agitation-associated mechanism. **b**1 is for TIG and **b**2 is for PPG

The effects of agitation on metabolic distribution in *C. acetobutylicum* are summarized in Fig. 8a. Based on our findings, we propose a possible mechanism by which *C. acetobutylicum* cells respond to agitation.

The high mixing efficiency in PPG enhances the PPP flux, providing more precursors for nucleotide synthesis and promoting both cell growth and butanol tolerance. Furthermore, the high reducing power and increased carbon flux promote butanol biosynthesis. By contrast, traditional agitation leads to higher levels of hydrodynamic damage on cells. To resist shear forces, cells increase the levels of unsaturated fatty acids and amino acids to modulate membrane fluidity. To achieve this, cells rearrange their metabolism towards enhanced ATP synthesis, which results in enhancing the flux through glycolysis and acid synthesis at the expense of the pentose phosphate pathway. This effect of hydrodynamic damage is intensified by the chemotropic effects of solvents on the cell membrane.

In conclusion, the enormous differences impacted by agitation obviously associated with three issues which result in significant changes in cell metabolic behaviors: first, a rebalanced redox status; second, the energy (ATP) acquirement and consumption; third, the tolerance mechanism of the cell for survival of solvent (homeoviscous adaptation) [31, 36, 37].

Based on such key information, rational enhancement strategies were applied: (1) for TIG, we stopped agitation during the solvent—producing phase (60–120 h) and supplemented the pool available amino acids including 5 mM of Ile, Val, Trp, Phe, Met at 60 h; finally, butanol and the total solvent (acetone-butanol-ethanol) were increased by 65 % and 101 % to 11.65 g/L (2.7 g/g biomass) and 22.21 g/L (4.9 g/L biomass), respectively (as shown in Fig. 8b1); (2) for PPG, we supplemented the depleted amino acids during the solvent—producing phase (60–120 h) in PPG, including 5 mM Asp, Pro and Ile. In the end, butanol and the total solvent (acetone-butanol-ethanol) were increased by 9.7 and 11 % to 13.92 g/L (2.85 g/g biomass) and 26.32 g/L (5.38 g/g biomass), respectively (as shown in Fig. 8b2). This validation experiments provide us with the direct support for our supposition on agitation-associated mechanism. In the large scale production, we could supplement some raw materials (such as soybean products, mycoprotein products, meat, and bone meal et al.) which are cheap but rich in amino acids to control the overall cost. Besides, this rational conception of enhancing the engineering process by analyzing the metabolism bottleneck of the targeted product is efficient and inspiring, which would be very attractive when applied on some high-value products.

## Conclusion

This paper proposed a novel agitation type (periodic-peristole) and studied the association mechanism between cell metabolism and agitation (traditional and periodic-peristole agitation) using CFD and MFA technologies. As a new agitation type, periodic-peristole showed difference on the hydrodynamic characteristics from the tradition agitation. Among the characteristics, eddy lengthy can well scale the shear force damage of agitation on the cells. Furthermore, agitation exerts influence on the cell flux distribution, which is highly associated with intercellular redox and energy status. This agitation-associated mechanism can guide the way for rational enhancement of the biofuel refinery process.

## Methods

### Microorganism and culture conditions

The working strain *Clostridium acetobutylicum* ATCC 824 was purchased from China General Microbiological Culture Collection Center and repetitively domesticated using the method of Yu et al. [47]. The fermentation method was described in our previous work [48]. There are three fermentation groups in this paper: periodic-peristole agitation group (PPG), traditional *Rushton impeller* agitation group (TIG), and stationary culture group (SG). Fermentation experiments in SG were performed in a 4 L BIOTECH-3BH (New Brunswick Scientific, USA) with a working volume of 1.8 L at 37 °C. The stirring speed in this group was set at 0 rpm; In TIG fermentation was also performed in 4 L BIOTECH-3BH (New Brunswick Scientific, USA) with a working volume of 1.8 L, 37 °C and the stirring speed was set at 55 rpm in the comparison fermentation experiment. The bioreactor configuration with periodic-peristole agitation is given in Additional file 1: Figure S1. The lower half bioreactor wall can shrink and extend periodically (just as stomach and intestine do) at set speed. In our research, the working volume of the bioreactor was 1.8 L with a total reactor volume of 4.0 L. The shrinkage rate was set at 0.5 cm/s in the comparison fermentation experiment. All experiments were carried out five times to ensure the reproducibility.

### Intracellular amino acid sampling and quantification

Because of the rapid turnover of intracellular metabolites, the sampling and quenching processes must be carried out in a rapid and reproducible manner to ensure proper quenching of intracellular amino acids and little amino acid loss related to leakage. The intracellular metabolites were extracted and measured as described previously [23]. D-sorbitol-^13^C6 (St. Louis, MO, USA) was added to 100 μL extract as internal standard for analysis. Five biological replicates were used to perform multivariate analysis for each sample.

### Analysis of substrate and products

Culture broth samples were taken every 12 h for measurement. The biomass was determined with the method of Jiang et al. [14]. Lactate, butyrate, butanol, acetone, ethanol, pyruvate, glucose were measured following the method of Wang, Chen [49]. NADH, NAD^+^, NADPH, NADP^+^, and ATP were measured using the method of Amador-Noguez et al. [50]. Hydrogen and carbon dioxide were measured with the method of Li, Chen [51]. Fatty acids were measured using the method of Xia et al. [23]. All the authentic standards were purchased from Sigma–Aldrich.

### Computational fluid dynamics (CFD) modeling

Force analysis was carried out in ANASYS Fluent (Version 14.5, ANASYS, NH, USA). The bioreactor geometry was incorporated into the commercial CFD software CFX 11.0 (ANSYS Inc., Canonsburg, PA, USA). The bioreactor geometry was given in Table 1. The fluid was simplified as the uniform liquid phase with the same viscosity value (1.3 × 10^−3^ Pa s). Mixing time was measured by the conductivity method using saturated sodium chloride solution as the tracer [52]. Eddy length was calculated followed the Kolmogorov’ model, which is following the expression of Eq. 1.

1 $${\text{Eddy lengh}} = \left[ {\frac{{(\frac{\mu }{\rho })^{3} }}{Pm}} \right]^{1/4}$$

where, μ is the dynamic viscosity, which is 1.3 × 10^−3^ Pa s in our study; ρ is the density of the fluid, which is 1010 kg/m^3^ in our study; and Pm is power input into the system per unit mass of fluid (W/kg);

### Determination of the relationship between shear force and cell growth

The experiment method to determine the shear force and cell growth was carried out as following: first, ten reactors with periodic-peristole agitation (Group I) and ten reactors with tradition agitation (group II) were set at different agitation rates to generate different shear forces (eddy length ranges from 60 to 180 μm) within each group; a reactor without agitation was set as control (Group III); second, *Clostridium acetobutylicum* strain was cultivated in the bioreactors with same cultivation condition, which was described in our previous work [45] for 120 h; at least, $\frac{{{\text{Bio}}_{\text{experimental}} }}{{{\text{Bio}}_{\text{control}} }}$ was calculated using the biomass data. The experiment result was shown in Fig. 3a.

### Metabolic flux analysis

A detailed network model for *C. acetobutylicum* metabolism was constructed on the basis of the batch fermentation data and pathway information collected from other studies reported in the literature, which employed species of *Clostridium* [13–16] and the KEGG Pathway Database (http://www.kegg.jp/). The network model contains 35 intracellular metabolites and 33 metabolic reactions in the glycolysis, pentose phosphate pathway (PPP), TCA cycle, and the biomass synthesis reactions. The cellular composition of *C. acetobutylicum* was assumed to be the same value as reported for *Clostridia* by Cai et al. [13] Biomass formation (growth flux) was included into the model to account for the drain of precursors and building blocks into biomass. A list of these selected reactions and metabolite abbreviations is provided in Additional files 4 and 5.

In this experiment, the specific rate for glucose uptake and the specific formation rates of lactate, acetate, butyrate, ethanol, CO_2_, H_2_, and acetone were used as the constraints in the Metabolic flux analysis (MFA) model. All the flux distributions were normalized by the glucose uptake rate on a basis of 100 mmol/(g cell h) and expressed as percentage.

### Data processing and statistical analysis

PLS-discriminant analysis (PLS) was applied to the data after mean-centering [53] on SIMCA package (Ver 10.0, Umetrics, Umea, Sweden). The generation rate of metabolites, cell growth rate and glucose utilization rate were set as input and butanol production rate was set as output. The analyses employed a default sevenfold internal cross validation [23]. For comparison the *Student’s t*-*test* was also carried out and the difference can be treated as significant when p < 0.05.

## Additional files

10.1186/s13068-015-0409-6 The two agitation types. (A), the agitation with a traditional *Rushton* impeller. (B), the periodic-peristole agitation.
10.1186/s13068-015-0409-6 The metabolite profiles comparisons between periodic-peristole group (PPG), the traditional Rushton impeller group (TIG); and the stationary group (SG) by student’s t-test.
10.1186/s13068-015-0409-6 Time course profiles of hydrogen production and carbon dioxide from PPG, TIG and SG. PPG represents periodic-peristole group, TIG represents traditional Rushton impeller agitation group, and SG represents stationary culture group. The values shown represent the means of five independent experiments and the error bars represent standard deviations of five values.
10.1186/s13068-015-0409-6 The abbreviations of the metabolite compounds.
10.1186/s13068-015-0409-6 Reactions used in metabolic flux model of *Clostridium acetobutylicum* ATCC 824.
10.1186/s13068-015-0409-6 PLS analysis for the data on biomass and total solvent.
10.1186/s13068-015-0409-6 Concentrations of intracellular amino acids in different agitation model during 0~120 h (Fig. 7c, d).

## Abbreviations

PPG: periodic-peristole group
TIG: the traditional Rushton impeller group
TIG: the stationary group (SG)
AKG: α-ketoglutarate
OAA: oxaloacetate
BA: butyrate
Eth: ethanol
LA: lactate
AA: acetate
AcCoA: acetyl-CoA
X5P: xylulose-5-phosphate
S7P: d-Sedoheptulose-7-phosphate
E4P: d-erythrose-4-phosphate
FDP: d-fructose 1, 6-bisphosphate
G6P: glucose 6-phosphate
ICIT: isocitrate
CIT: citrate
PYR: pyruvate
PEP: phosphoenolpyruvate
2GP: 2-phospho-d-glycerate
1, 3GP: 1, 3-phospho-d-glyceroyl phosphate
3GP: 3-phospho-d-glycerate
GAP: glyceraldehyde-3-phosphate
R5P: d-ribose-5-phosphate
ACE: acetone
D6PAH3U: d-arabino-6-Phospho-hex-3-ulose
Ru5P: d-Ribulose 5-phosphate
Glu: glutamate
Ala: alanine
Asp: aspartate
Pro: proline
Gly: glycine
Ser: serine
Leu: leucine
Ile: isoleucine
Val: valine
Trp: tryptophan
Phe: phenylalanine
Met: methionine
