# Supplementary material for: Periodic-peristole agitation for process enhancement of butanol fermentation
Source: Biotechnol Biofuels. 2015 Dec 23;8:225. doi: 10.1186/s13068-015-0409-6 (PMC4689062; doi:10.1186/s13068-015-0409-6)
Supplement: Supplementary file 1 — 10.1186/s13068-015-0409-6 The two agitation types. (A), the agitation with a traditional Rushton impeller. (B), the periodic-peristole agitation. [file 13068_2015_409_MOESM1_ESM.pdf]

Supplementary I

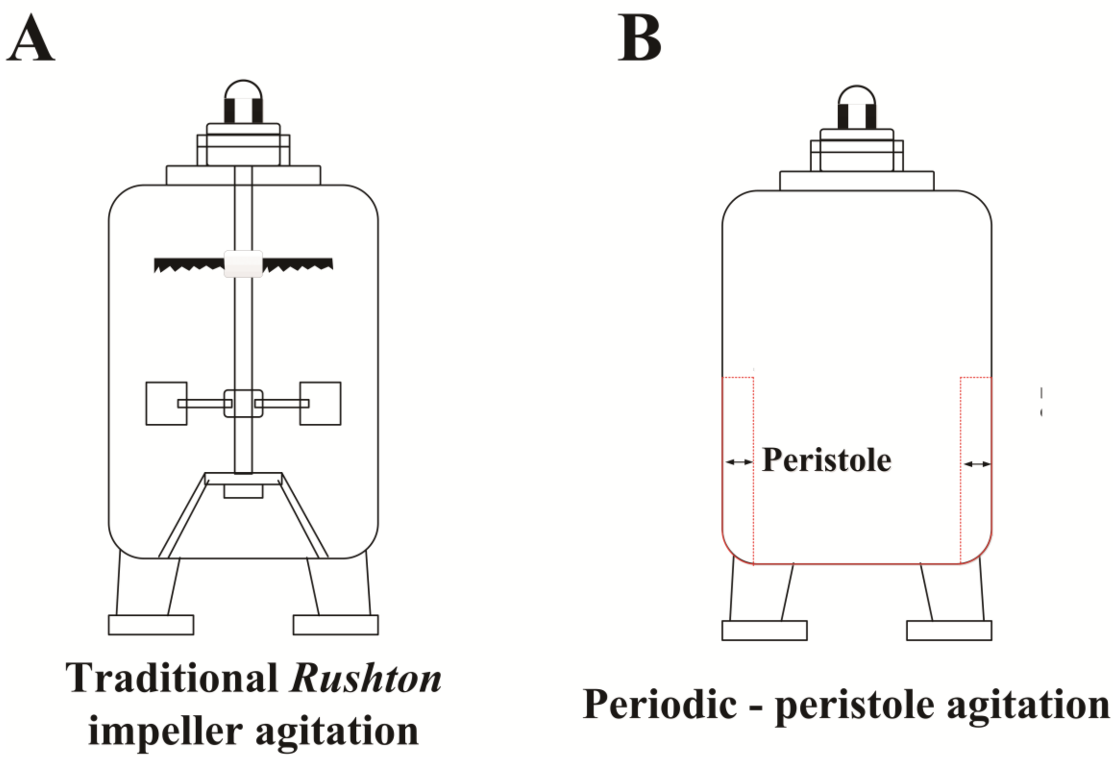

Figure S-1 the two agitation types. (A), the agitation with a traditional *Rushton* impeller. (B), the periodic - peristole agitation.
