# Supplementary material for: Periodic-peristole agitation for process enhancement of butanol fermentation
Source: Biotechnol Biofuels. 2015 Dec 23;8:225. doi: 10.1186/s13068-015-0409-6 (PMC4689062; doi:10.1186/s13068-015-0409-6)
Supplement: Supplementary file 2 — 10.1186/s13068-015-0409-6 The metabolite profiles comparisons between periodic-peristole group (PPG), the traditional Rushton impeller group (TIG); and the stationary group (SG) by student’s t-test. [file 13068_2015_409_MOESM2_ESM.pdf]

Supplementary II

Table S-1 the metabolite profiles comparisons between periodic - peristole group (PPG), the traditional Rushton impeller group (TIG); and the stationary group (SG) by student's t-test.

Materials	P values		
	PPG-SG*	PPG-TIG*	SG-TIG*
Biomass	0.00051	0.0016	0.89
Glucose utilization	0.00025	0.00072	0.15
Acetone production	0.00011	0.00019	0.00085
Butanol	0.0067	0.0107	0.0187
Butyrate	0.00062	0.10	0.019
Lactic acid	0.48	0.00036	0.000032
Ethanol	0.00027	0.00055	0.0014
Acetic acid	0.11	0.00026	0.00093
Hydrogen	0.00020	0.00080	0.021

Note: PPG-SG represents: the student's t-test between PPG and SG; PPG-TIG represents: the student's t-test between PPG and TIG; SG-TIG represents: the student's t-test between SG and TIG;
