# Supplementary material for: Periodic-peristole agitation for process enhancement of butanol fermentation
Source: Biotechnol Biofuels. 2015 Dec 23;8:225. doi: 10.1186/s13068-015-0409-6 (PMC4689062; doi:10.1186/s13068-015-0409-6)
Supplement: Supplementary file 3 — 10.1186/s13068-015-0409-6 Time course profiles of hydrogen production and carbon dioxide from PPG, TIG and SG. PPG represents periodic-peristole group, TIG represents traditional Rushton impeller agitation group, and SG represents stationary culture group. The values shown represent the means of five independent experiments and the error bars represent standard deviations of five values. [file 13068_2015_409_MOESM3_ESM.pdf]

Supplementary III

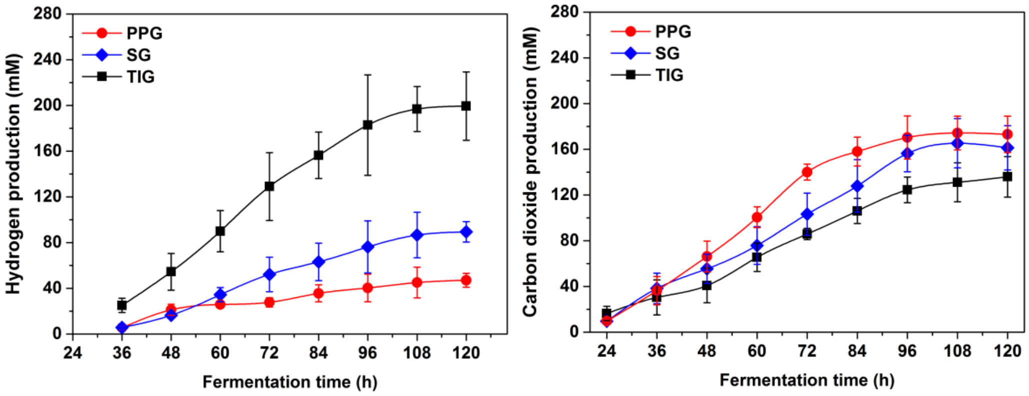

Figure S-2. Time course profiles of hydrogen production and carbon dioxide from PPG, TIG and SG. PPG represents periodic - peristole group, TIG represents traditional *Rushton* impeller agitation group, and SG represents stationary culture group. The values shown represent the means of five independent experiments and the error bars represent standard deviations of five values.
