# Supplementary material for: Periodic-peristole agitation for process enhancement of butanol fermentation
Source: Biotechnol Biofuels. 2015 Dec 23;8:225. doi: 10.1186/s13068-015-0409-6 (PMC4689062; doi:10.1186/s13068-015-0409-6)
Supplement: Supplementary file 4 — 10.1186/s13068-015-0409-6 The abbreviations of the metabolite compounds. [file 13068_2015_409_MOESM4_ESM.pdf]

Supplementary IV. The abbreviations of the metabolite compounds

Abbreviation	Compound
1,3PG	3-phospho-D-glyceroyl phosphate
2 PG	2-phospho-D-glycerate
3 PG	3-phospho-D-glycerate
AA	Acetate
AcCoA	Acetyl CoA
Ace	Acetone
AKG	A-ketoglutarate
BA	Butyrate
BB	Butanol
Bio	Biomass
CIT	Citrate
CO2	Carbon dioxide
D6PAH3U	D-arabino-6-Phospho-hex-3-ulose
DHAP	Dihydroxyacetone phosphate
E4P	D-erythrose 4-phosphate
ETH	Ethanol
F6P	D-fructose-6-phosphate
FDP	D-fructose 1, 6-bisphosphate
Fd	Ferredoxin
CH2O	Formaldehyde
FdH2	Reduced ferredoxin
G6P	Glucose 6-phosphate
GAP	Glyceraldehyde 3-phosphate
Glc	Glucose
ICIT	Isocitrate
LA	Lactate
NADH	Reduced-nicotinamide adenine dinucleotide
NADPH	Reduced-nicotinamide adenine dinucleotide phosphate
OAA	Oxaloacetate
PEP	Phosphoenolpyruvate
PYR	Pyruvate
R5P	D-ribose-5-phosphate
Ru5P	D-ribulose-5-phosphate
S7P	D-sedoheptulose-7-phosphate
X5P	Xylulose-5-phosphate
