# Supplementary material for: Periodic-peristole agitation for process enhancement of butanol fermentation
Source: Biotechnol Biofuels. 2015 Dec 23;8:225. doi: 10.1186/s13068-015-0409-6 (PMC4689062; doi:10.1186/s13068-015-0409-6)
Supplement: Supplementary file 5 — 10.1186/s13068-015-0409-6 Reactions used in metabolic flux model of Clostridium acetobutylicum ATCC 824. [file 13068_2015_409_MOESM5_ESM.pdf]

Supplementary V

Reactions used in metabolic flux model of *Clostridium acetobutylicum* ATCC 824

Embden–Meyerhof-Parnas pathway (EMP)

r1 Glc+PEP→G6P+Pyr
r2 G6P↔F6P
r3 F6P↔FDP
r4 FDP↔DHAP+GAP
r5 DHAP↔GAP
r6 GAP↔1,3PG+NADH
r7 1,3PG↔3PG
r8 3PG↔2PG
r9 2PG↔PEP
r10 PEP→PYR

Pentose phosphate pathway

r11 F6P ↔D6PAH3U
r12 D6PAH3U→Ru5P+ CH2O
r13 Ru5P↔R5P
r14 Ru5P↔X5P
r15 R5P+X5P↔GAP+S7P
r16 E4P+X5P→F6P+GAP
r17 GAP+S7P→E4P+F6P

Pyruvate metabolism

r18 NADH+PYR↔LA
r19 PYR→AcCoA+FdH2+CO2
r20 AcCoA→AA
r21 AcCoA+2NADH↔ETH
r22 2AcCoA+2NADH→BA
r23 2AcCoA+4NADH→BB
r24 AcCoA +AA + NADH→ ACE +CO2

Hydrogen formation

r25 NADH↔FdH2
r26 FdH2↔H2

TCA cycle

r27 PEP+CO2↔OAA
r28 AcCoA+OAA→CIT
r29 CIT↔ICIT
r30 ICIT↔AKG+NADH
r31 OAA+PYR→AKG+NADH+NADPH+2CO2

Transhydrogenation reaction

r32 NADPH↔NADH

Biomass formation

r33 1.239 3PG+1.01 AcCoA + 0.6285 AKG + 0.297 E4P + 0.1788 F6P + 7.6152 NADPH + 1.4452 OAA + 0.6411 PEP + 1.6666 PYR + 0.5726 R5P → Bio + 1.31685 NADH
