# Supplementary material for: Periodic-peristole agitation for process enhancement of butanol fermentation
Source: Biotechnol Biofuels. 2015 Dec 23;8:225. doi: 10.1186/s13068-015-0409-6 (PMC4689062; doi:10.1186/s13068-015-0409-6)
Supplement: Supplementary file 6 — 10.1186/s13068-015-0409-6 PLS analysis for the data on biomass and total solvent. [file 13068_2015_409_MOESM6_ESM.pdf]

Supplementary VI

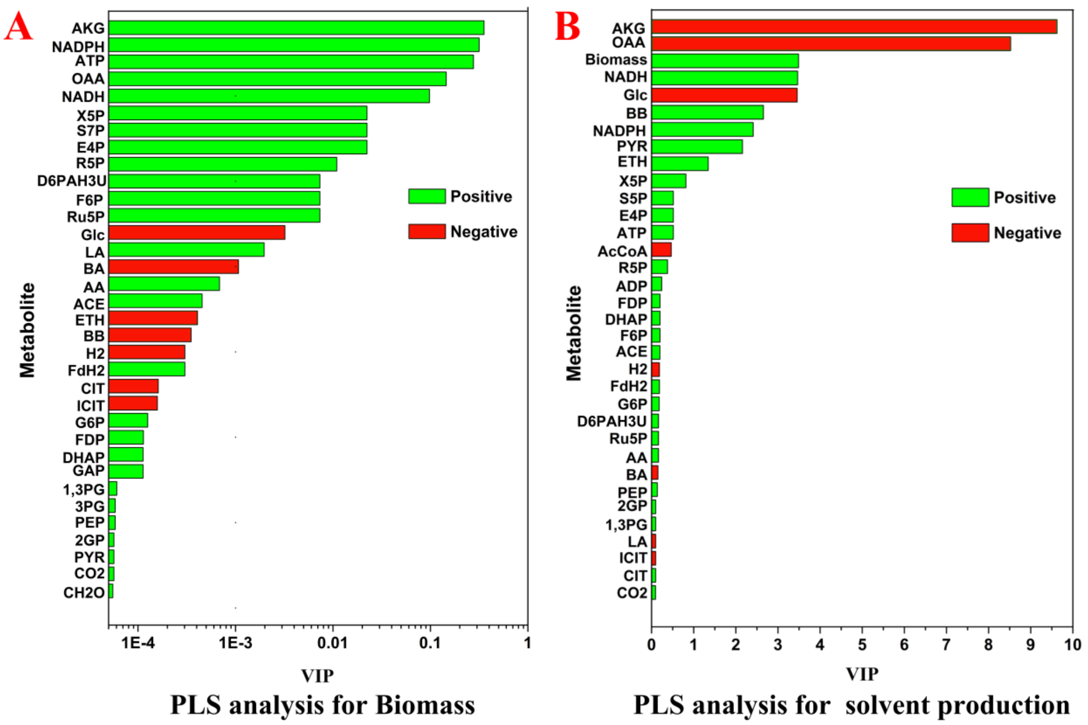

Figure S-3 PLS analysis for the data on biomass and total solvent. Figure S-3A showed the PLS analysis results on biomass (0~60 h, the cell growth phase). Figure S-3B showed the analysis results on solvents (ethanol-acetone-butanol) production.

PLS analysis on Biomass

As Figure S-2A shown, metabolites from *Pentose phosphate pathway* (PPP) (including X5P, S7P, E4P, R5P, and Ru5P) were determined to be important for cell growth, because PPP serves the main source of ribose, the sugar backbone of nucleotides, for cell growth [1]. This result was consistent with the analysis in the manuscript.

TCA metabolism was also listed as the most important pathway for biomass synthesis. This might mainly because TCA contributed precursors and ATP for amino acids synthesis during cell growth (0~60 h) [2].

PLS analysis on Solvent

As Figure S-2A shown, metabolites exhibited similar relationship with solvent and

butanol. What should be noted is that TCA (AKG and OAA) played opposite roles in solvent production (60~120h). It can be explained by the study of Ezeji T [3]: cells in solvent producing phase ceased to grow in the solvent-producing phase and should distribute the flux of TCA and PPP towards butanol synthesis. High flux among TCA in the solvent producing phase would compete for carbon flux, ATP and reducing power with butanol synthesis. Therefore, VIP coefficients of AKG and OAA were negative for solvent production. This had been discussed in the manuscript.

Reference

1. Tian WN, Braunstein LD, Pang J, Stuhlmeier KM, Xi Q-C, Tian X et al. Importance of glucose-6-phosphate dehydrogenase activity for cell growth. J Biol Chem. 1998, 273(17):10609-17.

2. Korneli C, Bolten CJ, Godard T, Franco-Lara E, Wittmann C. Debottlenecking recombinant protein production in *Bacillus megaterium* under large-scale conditions-targeted precursor feeding designed from metabolomics. Biotechnol Bioeng. 2012, 109(6):1538-50. Doi:Doi 10.1002/Bit.24434.

3. Ezeji T, Milne C, Price ND, Blaschek HP. Achievements and perspectives to overcome the poor solvent resistance in acetone and butanol-producing microorganisms. Appl Microbiol Biotechnol 2010, 85(6):1697-712.
