# Supplementary material for: Periodic-peristole agitation for process enhancement of butanol fermentation
Source: Biotechnol Biofuels. 2015 Dec 23;8:225. doi: 10.1186/s13068-015-0409-6 (PMC4689062; doi:10.1186/s13068-015-0409-6)
Supplement: Supplementary file 7 — 10.1186/s13068-015-0409-6 Concentrations of intracellular amino acids in different agitation model during 0~120 h (Fig. 7c, d). [file 13068_2015_409_MOESM7_ESM.pdf]

Supplementary VII

Table S-2. The concentration of intracellular amino acids in Periodic - peristole agitation group during 0 ~120 h

	**12 h**	**24 h**	**36 h**	**48 h**	**60 h**	**72 h**	**84 h**	**96 h**	**108 h**	**120 h**
Glutamate	603.2 ±36.2	561.2 ±129.1	364.3 ±58.3	477.2 ±57.3	565 ±62.2	615.9 ±73.9	721.3 ±129.8	757.1 ±30.3	804.7 ±72.4	682.3 ±143.3
Alanine	148.9 ±10.4	130.8 ±11.8	133 ±5.3	137.1 ±8.2	139.5 ±18.1	121.7 ±15.8	136.3 ±17.7	135.6 ±10.8	134.8 ±5.4	141 ±14.1
Aspartate	21.1 ±0.6	22.7 ±1.6	19.3 ±4.6	17.8 ±0.5	16.3 ±0.7	16.2 ±3.2	24.3 ±5.6	28.2 ±1.1	22.2 ±2	22.7 ±2
Proline	14.3 ±2.4	16 ±2.4	15.3 ±2.9	15.9 ±3.8	11.8 ±0.8	11.8 ±2.5	6.5 ±0.3	5 ±0.7	5.1 ±0.2	3.8 ±0.8
Glycine	28.5 ±2.6	24.8 ±4.2	13.8 ±2.6	24.5 ±2.2	28.3 ±5.1	33 ±1.7	33.3 ±6.7	33.6 ±1	31.7 ±4.4	24.5 ±0.7
Serine	2.5 ±0.4	3 ±0.4	3.2 ±0.1	4 ±0.4	4 ±0.4	3.4 ±0.2	3.4 ±0.6	3.6 ±0.8	2.8 ±0.6	3.4 ±0.1
Threonine	6.4 ±1.2	6.2 ±0.4	6.3 ±1.4	7 ±0.7	7.1 ±1.6	7.7 ±0.8	9.2 ±1.4	9 ±0.6	10.3 ±1.8	8.7 ±1.6
Leucine	1.2 ±0.2	1.3 ±0.3	1.8 ±0.4	1.7 ±0.2	2 ±0.1	2.1 ±0.1	1.9 ±0.1	1.9 ±0.1	1.6 ±0.1	1.1 ±0.2
Isoleucine	7.8 ±1.2	12.7 ±0.5	11.8 ±3	11.2 ±1.9	13.4 ±3.4	12.5 ±1.9	11.3 ±1.8	9.7 ±1	8.3 ±0.3	2.1 ±0.3
Valine	10.5 ±1.4	12.1 ±0.6	12.7 ±2.8	14.4 ±0.4	16.2 ±2.4	17.9 ±1.8	19 ±1.7	19.1 ±2.5	20.3 ±4.1	18.7 ±3.6
Tryptophan	11.3 ±0.6	12.3 ±0.7	13.9 ±2.9	14.6 ±3.2	14.9 ±2.8	16.6 ±1.2	15.3 ±0.9	15.4 ±0.8	15.8 ±3.6	16.6 ±3.2
Phenylalanine	1.4 ±0.2	1.3 ±0.1	1.5 ±0.2	1.7 ±0.3	1.6 ±0.4	1.6 ±0.1	1.7 ±0.1	1.8 ±0.5	1.6 ±0.2	1.7 ±0.3
Methionine	4.5 ±1	6.2 ±1.1	5.8 ±0.4	5.5 ±1.2	5.7 ±0.5	6.1 ±1.4	6.6 ±1.5	5.8 ±0.6	4.9 ±0.2	3.6 ±0.3
Histidine	2.8±1.0	4.5±2.8	5.2±1.5	4.9±1.3	4.0±2.6	4.7±1.5	5.5±2.0	3.7±1.3	2.1±1.1	0.7±0.2

All concentrations are given in μmol (g cell dry weight)$^{-1}$.

Table S-3. The concentration of intracellular amino acids in traditional *Rushton* impeller agitation group during 0 ~120 h

	**12 h**	**24 h**	**36 h**	**48 h**	**60 h**	**72 h**	**84 h**	**96 h**	**108 h**	**120 h**
Glutamate	925.9 ±166.7	739.8 ±155.4	831.5 ±83.2	627.3 ±100.4	643.2 ±128.6	902.7 ±108.3	924 ±83.2	986.7 ±29.6	1144.9 ±114.5	1059.6 ±106
Alanine	124.8 ±18.7	131.7 ±25	127.6 ±23	119.4 ±25.1	120.5 ±6	118.4 ±21.3	121.9 ±3.7	119.7 ±14.4	135.4 ±13.5	101.7 ±6.1
Aspartate	37.9 ±4.5	45.1 ±1.8	43.6 ±8.7	48.2 ±5.3	48.6 ±4.4	45.7 ±9.1	47.5 ±8.5	51.6 ±9.3	55.1 ±4.4	52.4 ±8.9
Proline	22.7 ±0.9	26.8 ±1.1	27.2 ±4.4	28.2 ±2	27 ±2.2	22.4 ±2.7	19.2 ±2.3	18.1 ±3.4	11.7 ±0.9	9.8 ±2.5
Glycine	129.3 ±25.9	130.4 ±6.5	94 ±18.8	94.2 ±3.8	80.4 ±12.1	87.5 ±15.8	71.9 ±9.3	76.8 ±11.5	75.5 ±17.4	58.3 ±3.5
Serine	10.1 ±1	12.9 ±2.7	14.1 ±1.1	14 ±2.8	11.4 ±0.6	10.5 ±0.5	9.9 ±1.7	8.7 ±0.4	5 ±1	5.1 ±0.4
Threonine	14.9 ±2.4	15.5 ±3.7	15.8 ±3	17 ±1.2	14 ±1.7	13.1 ±3.1	12 ±0.5	11.5 ±2	10 ±1.5	5.8 ±0.7
Leucine	4.2 ±0.8	4.1 ±0.7	4.1 ±1	4 ±0.4	4 ±0.2	3.7 ±0.3	3.1 ±0.3	2.9 ±0.1	3 ±0.2	3.1 ±0.1
Isoleucine	11.2 ±0.6	10.7 ±0.6	9.7 ±2.1	8.2 ±1.2	8.4 ±0.4	6.7 ±0.6	8.3 ±1.7	3.2 ±0.2	0 ±0	0.4 ±0.1
Valine	3.1 ±0.2	3.9 ±0.7	3.3 ±0.1	5 ±0.4	6.7 ±1.4	9.4 ±2	9.7 ±1.8	10.4 ±0.5	10.3 ±0.4	7 ±0.8
Tryptophan	2.7 ±0.4	2.7 ±0.1	3.5 ±0.4	5.8 ±1	6.8 ±0.6	8 ±1.1	6.9 ±0.3	6.6 ±0.4	5.7 ±1.4	5 ±1.3
Phenylalanine	1.6 ±0.2	1.9 ±0.1	2.1 ±0.2	2.2 ±0.3	2 ±0.3	0.7 ±0.1	0.2 ±0	0.1 ±0	0 ±0	0.3 ±0
Methionine	5.6 ±1.3	7.4 ±1.3	6.4 ±1.2	8.1 ±0.5	7.3 ±1.8	7.2 ±0.9	1.4 ±0.1	0.5 ±0	0.2 ±0	0 ±0
Histidine	3.0±0.9	3.5±1.0	3.4±0.48	3.9±0.46	4.1±0.96	4.1±1.6	4.8±0.97	4.3±1.1	4.5±0.68	1.8±0.53

All concentrations are given in μmol (g cell dry weight)$^{-1}$.

Table S-4. The concentration of intracellular amino acids in stationary culture group during 0 ~120 h

	**12 h**	**24 h**	**36 h**	**48 h**	**60 h**	**72 h**	**84 h**	**96 h**	**108 h**	**120 h**
Glutamate	618 ±37.1	621.3 ±80.8	647.7 ±155.4	664 ±46.5	694.5 ±34.7	682.2 ±163.7	691.9 ±76.1	682.6 ±75.1	722.9 ±137.4	746.9 ±74.7
Alanine	124.1 ±13.7	124.1 ±14.9	122.5 ±15.9	123.9 ±7.4	123.4 ±18.5	123.5 ±25.9	123.1 ±25.9	122.7 ±30.7	122.1 ±19.5	120.7 ±21.7
Aspartate	30.9 ±1.9	30.8 ±3.4	32.9 ±2.6	35.1 ±6.3	35.3 ±3.5	34.2 ±5.5	33.5 ±3.4	33.2 ±2.7	32.5 ±2.3	31.8 ±3.5
Proline	9.5 ±1.9	10.2 ±1.5	10.2 ±2	9.8 ±2.3	9.8 ±1.5	9.9 ±1.3	10.2 ±2.1	10.1 ±1.8	8.9 ±2.2	7.4 ±1.3
Glycine	22.7 ±5.2	24 ±4.8	25.8 ±5.2	23.5 ±3.3	23.1 ±2.8	24.6 ±2	24.2 ±5.1	25.8 ±4.1	25 ±2.3	28 ±0.8
Serine	3.9 ±0.4	4.7 ±0.6	4.4 ±0.9	3.6 ±0.7	3.4 ±0.4	4 ±0.8	4.1 ±0.9	3.4 ±0.4	3.6 ±0.9	3.1 ±0.7
Threonine	0.6 ±0.1	1.1 ±0.1	1.3 ±0.3	1.1 ±0.1	1.4 ±0.1	1.9 ±0.2	1.7 ±0.2	1.9 ±0.1	1.2 ±0.1	1.2 ±0.3
Leucine	1.3 ±0.1	1.5 ±0.1	1.5 ±0.1	1.4 ±0.3	1.5 ±0.1	1.6 ±0.1	1.6 ±0.3	1.6 ±0	1.5 ±0.2	1.5 ±0.3
Isoleucine	2.9 ±0.4	3.7 ±0.1	4.1 ±0.7	3.9 ±0.6	3.6 ±0.1	4 ±0.8	3.9 ±0.9	3.8 ±0.5	3.5 ±0.2	3.2 ±0.7
Valine	15.4 ±3.4	15.4 ±1.4	15.9 ±2.1	15.6 ±2.8	15.1 ±3.6	14.4 ±2.6	14.1 ±1.8	13.9 ±1	13.7 ±2.3	13.8 ±1.5
Tryptophan	5.9 ±0.9	6.2 ±0.6	6.2 ±0.4	6 ±0.2	6.1 ±1.1	6.2 ±1.2	6.9 ±0.3	7.4 ±1.4	7.7 ±0.5	6.2 ±1.1
Phenylalanine	0.5 ±0.1	0.6 ±0.1	0.6 ±0	0.6 ±0.1	0.6 ±0.1	0.7 ±0.1	0.6 ±0.1	0.5 ±0.1	0.5 ±0	0.4 ±0.1
Methionine	4.6 ±0.4	4.7 ±1.2	4.8 ±1	4.7 ±0.9	4.6 ±0.3	4.4 ±0.5	4.3 ±0.7	4.3 ±0.9	4.3 ±0.8	4.3 ±0.6
Histidine	2.6±0.91	7.2±2.5	4.7±2.1	8.1±3.6	7.2±2.9	4.3±2.4	4.4±2.1	2.5±1.4	2.4±1.1	0.4±0.2

All concentrations are given in μmol (g cell dry weight)$^{-1}$.
